# Causes of Elective Surgery Procedure Cancellation in a Portuguese General Hospital: A Cross-Sectional Observational Study

**DOI:** 10.7759/cureus.117302

**Published:** 2026-10-01

**Authors:** Fernando Cunha, Inês Baptista, Maria Inês Andrade, Inês Castro Leal, Daniela Chalo

**Affiliations:** 1 Anesthesiology, Unidade Local de Saúde da Região de Aveiro, Aveiro, PRT; 2 Mathematics, University of Aveiro, Aveiro, PRT

**Keywords:** cancellation rate, causes of surgery postponement, elective surgery cancellation, operating theatre efficiency, pre-anaesthetic assessment, surgical specialties

## Abstract

Introduction: Elective surgery cancellation is a source of patient dissatisfaction and improper resource allocation, since it decreases the working efficiency of the operating theater and increases healthcare system costs. Knowing the causes behind cancellation can be helpful in establishing an action plan to prevent them from happening. This study aimed to quantify the rate and identify the main causes of elective surgery cancellation at a Portuguese general hospital, and to examine the relationship between cancellation causes and relevant clinical and organizational variables.

Materials and methods: Data regarding elective surgery cancellations from January to October 2023 were collected retrospectively. Details on the type of surgery, American Society of Anesthesiologists (ASA) physical status, pre-anesthetic assessment, and the specific reason for cancellation were gathered.

Results: During the period analyzed, 5284 elective surgeries were scheduled. The local cancellation rate was 12.24%. Most of the postponed patients attended the preoperative anesthetic evaluation (82.8%, n = 536). The five main causes identified were "strike," "lack of theater time," "patient-related factors-disease," "patient did not attend," and "patient-related factors-other reasons." The most frequent cause of cancellation was strike (54%, n = 350), followed by lack of theater time (23.3%, n = 152) and patient-related factors-disease (9.7%, n = 63). By specialty, most cases were cancelled in orthopedics (25.8%, n = 167), followed by general surgery (21.2%, n = 137).

Conclusion: Cancellations have an adverse effect on the concept of theater "efficiency." After the "strike," we found that "lack of theater time" was the most important modifiable cause for elective surgical cancellation. To define the processes that can be improved, an audit could be done, in the future, to assess problems of scheduling, overbooking, or theater over-utilization. Future directions could also include a more profound analysis of the impact of multidisciplinary preoperative anesthesia clinic evaluation on cancellation rates.

## Introduction

The cancellation of elective surgeries represents a significant source of patient dissatisfaction and inefficient resource utilization, as it decreases the operational efficiency of the operating theater (OT) and increases healthcare system costs [1,2]. Elective procedures may be cancelled or rescheduled.

Furthermore, cancellations may lead to emotional distress, repeated preoperative fasting, and additional financial burdens for patients, thereby compromising their overall satisfaction [3,4]. Improving patient satisfaction through efficient clinical practice is a key objective of modern healthcare systems; however, high rates of elective surgery cancellations hinder this goal [2,5,6].

The operational efficiency of a hospital is partly dependent on the performance of its OT. However, this performance is directly influenced by the coordination of other hospital departments, establishing a natural and dynamic interdependence that requires effective communication and management across services to achieve institutional and departmental objectives. The cancellation rate is considered an important quality indicator and is routinely monitored and reported by hospitals [6].

When cancellations occur on the day of surgery, the negative impact is twofold, as it is often impossible to efficiently reschedule another patient to occupy the available time slot. This situation results in operational and financial losses, underutilization of operating rooms, and the waste of specialized human resources, materials, and equipment. These events directly affect institutional productivity and overall efficiency [7].

Regardless of timing or cause, the Ministry of Health provides hospitals with specific guidelines regarding the non-performance of scheduled surgeries. When cancellations occur for reasons beyond the patient's control--either clinical or operational--it is recommended that the scheduling, rather than the surgery itself, be cancelled, allowing the patient to remain eligible for rescheduling. In such cases, it is further recommended to call a substitute patient to fill the available OT slot [8].

The causes of surgical cancellations are highly variable and may be grouped into several categories. The Sistema Integrado de Gestão de Inscritos para Cirurgia (SIGIC) 5 Manual (Integrated Management System for Surgical Waiting Lists) divides them into four major groups: (a) Patient responsibility for non-clinical reasons (refusal with or without justification, absence with or without justification, withdrawal, or inability to contact the patient); (b) Clinical reasons (death or other medical causes); (c) Hospital responsibility (inability of the OT or inpatient wards to respond, administrative error, loss of surgical capacity--such as transfer due to exceeding the maximum guaranteed waiting times); and (d) Third-party responsibility (surgery performed in another institution, strike, etc. [7,9]). Strike is legally defined, under Portuguese labor law (Constitution, Article 57; Labor Code, Articles 530-543), as a concerted, collective withdrawal of labor by workers to defend a common occupational interest, subject to a mandatory minimum-services regime for essential care. Therefore, in this study, "strike" refers to the cancellation of elective procedures resulting from the collective withdrawal of labor by healthcare professionals (physicians and/or nursing staff) exercising their constitutionally protected right to strike.

Elective surgery cancellation is typically a multifactorial phenomenon, with many interrelated causes and a lack of standardized coding systems, which complicates detailed analysis and data interpretation [7]. The literature reports wide variability in cancellation rates and causes [4,10,11]. Commonly cited reasons include medical factors (e.g., incomplete preoperative assessment or treatment), OT-related factors (e.g., insufficient capacity, shortage of materials or personnel), and administrative issues (e.g., scheduling errors) [1].

In a recent systematic review of same-day surgical cancellations, Al Talawah and McIltrot [12] highlighted the considerable variability in cancellation rates across studies, even among hospitals with comparable size and case mix. This variability may stem from factors such as hospital type and size (degree of specialization, university affiliation), study methodology (grouping of cancellation causes), and differences in healthcare systems and policies. Institution-specific variables also play a crucial role, including surgical specialty, OT management model (flexible vs. fixed scheduling), and the implementation of processes aimed at minimizing cancellations, such as preoperative consultations [7,13].

A comparative retrospective study by Schuster et al. [11] found that cancellation rates were significantly higher in university hospitals and in the specialty of general surgery. Reported rates also vary substantially between countries: China (<1%), United States (2-12%), United Kingdom (5-14%), Spain (7%), Hong Kong (8%), Canada (10%), Australia (12%), and India (17%) [10]. Another study reported a range of 2-40% in high-income countries, with rates reaching up to 73% in low- and middle-income countries [10]. These discrepancies highlight the influence of contextual healthcare factors on surgical cancellations.

The classification of cancellation causes also varies across studies. Al Talawah and McIltrot categorized them as hospital-related (notably lack of operative time), patient-related, and surgeon/anesthesiologist-related (shortage of staff), whereas Schuster et al. grouped them as administrative/patient-related (most commonly absenteeism), clinical (changes in health status or poor preparation), and organizational (notably overbooking). Despite methodological differences, both authors concluded that most cancellations are institution-related [11,12].

While the global impact of elective surgery cancellations is well-documented, recent empirical data regarding cancellation rates, specialty-specific drivers, and rescheduling dynamics within Portuguese public hospitals remain limited. Regional healthcare networks, such as the Local Health Unit of the Aveiro Region (Unidade Local de Saúde da Região de Aveiro (ULSRA)), operate under specific structural workflows and national waiting-list management systems (SIGIC). However, robust local evidence evaluating operational efficiency and the impact of systemic disruptions remains lacking. To address this evidentiary gap, the primary aim of this study is to provide regional baseline data on elective surgery cancellations at ULSRA, identifying their primary clinical and operational causes to better design local healthcare policies and improve operating theater efficiency.

Specific objectives included: (1) Quantifying the rate of elective surgery cancellations. (2) Identifying the main causes of cancellation. (3) Examining the relationship between different variables, such as surgical specialty, sociodemographic parameters, reasons for cancellation, preoperative anesthetic consultation attendance, American Society of Anesthesiologists (ASA) physical status, and rescheduling interval.

## Materials and methods

Data

This study was designed as a cross-sectional observational study utilizing retrospective data encompassing all cancellations of elective (scheduled) surgeries conducted between January 1 and October 30, 2023.

Data were collected retrospectively from the hospital's operating room record system (BS PaTIENT. CARE) and general clinical records (SClínico) by resident physicians from the Department of Anesthesiology and entered into a Microsoft Excel (Microsoft Corporation, Redmond, Washington, USA) spreadsheet for subsequent analysis. Cases were excluded if there were duplicate entries or data inconsistencies, such as patients who underwent surgery on or before the scheduled date, or procedures not yet scheduled in the operating room system.

The sample size was determined by feasibility, capturing all eligible scheduled elective surgeries and confirmed cancellations registered at ULSRA across the entire 10-month study window.

This cross-sectional observational study evaluated all elective (scheduled) surgical procedures registered in the operating room management system at the Local Health Unit of the Aveiro Region (ULSRA) between January 1 and October 30, 2023.

The selection process followed a structured two-stage screening approach: 1. First, all scheduled elective procedures during the 10-month study period were identified to establish the total institutional surgical volume; n = 5284. 2. From this total population, cases flagged as non-performed/cancelled were extracted into an initial database of potential cancellations; n = 671. 3. Retrospective chart reviews were conducted by resident anesthesiologists to verify cancellation status. Cases were excluded if they met any of the following pre-defined criteria: duplicate database entries (n = 1) or operational/data inconsistencies (n = 23); defined as procedures completed on or prior to the scheduled date, or procedures not yet formally booked in the operating theater system. 4. Following exclusions, the final analyzed cohort comprised 647 confirmed cancelled elective surgeries.

Therefore, the inclusion criteria comprised all elective (scheduled) surgical procedures officially registered in the operating room management system at the Local Health Unit of the Aveiro Region (ULSRA) between January 1 and October 30, 2023, that were subsequently postponed, rescheduled, or cancelled within 48 hours of the scheduled date or on the day of surgery itself. Conversely, cases were excluded if they involved duplicate database entries, procedures completed on or prior to the scheduled date, procedures not yet formally booked in the operating theater system, or entries with operational and data inconsistencies.

Institutional approval and ethical considerations

The study protocol and data collection were formally approved by the Direction of the Anesthesiology Department and by the Hospital's Clinical Department. Because this was a retrospective study utilizing fully anonymized data without patient identifiers, individual ethical committee registration was waived in accordance with institutional guidelines.

Measures

The variables collected included: age, gender (male or female), surgical specialty, reason for cancellation, ASA physical status, scheduled intervention date, preoperative anesthetic consultation (yes or no), date of consultation, subsequent preoperative consultation (yes or no), date of the subsequent consultation, and, for surgeries that were rescheduled, the rescheduling date. An additional variable was created to calculate the number of days of delay between the initially scheduled surgery date and the rescheduled date (when applicable). The rescheduling interval was defined as the number of days between the initially scheduled and rescheduled surgery dates. For patients without a formal preoperative anesthetic consultation, the ASA classification was retrospectively assigned by the data-collecting anesthesiology residents based on the ASA classification registered by the attending anesthesiologist on the day of surgery.

Elective surgery cancellation was defined as any scheduled elective procedure that was officially booked on the operating theater (OT) schedule but was subsequently postponed, rescheduled, or cancelled within 48 hours of the scheduled date or on the day of surgery itself.

The surgical specialties considered were stomatology, general surgery, vascular surgery, gynecology, ophthalmology, orthopedics, otorhinolaryngology, and urology. Due to the heterogeneity observed in patient ages, these were grouped into five categories: 1-4 years, 5-12 years, 13-65 years, 66-85 years, and over 85 years.

These intervals were based on clinical and physiological criteria to differentiate distinct perioperative risk groups, specifically distinguishing pediatric, adult, elderly, and very elderly populations.

Analysis strategy

Statistical analysis was performed using R software (version 4.2.2) and RStudio (version 2023.03.0+386) (R Foundation for Statistical Computing, Vienna, Austria). The following packages were used: readxl, gtsummary, skimr, janitor, ggplot2, ggthemes, dplyr, agricolae, car, rcompanion, and dunn.test. The calculated p-values were associated with a 5% significance level; that is, a p-value lower than 0.05 was considered statistically significant.

Descriptive statistics were performed, followed by inferential analysis, which included the application of several statistical tests, namely the Pearson’s chi-square test, the Kruskal-Wallis test, the Wilcoxon-Mann-Whitney test, and Dunn's post-hoc test.

A Pearson's chi-square test was performed to assess the existence of a statistically significant association between surgical specialty and surgery cancellation, that is, whether the distribution of surgical cancellations is independent of specialty. The following hypotheses were formulated:

H_0_: There is no significant association between the two variables.

H_1_: There is a significant association between the two variables.

The associated p-value was less than 0.001, indicating statistical significance. Thus, there is sufficient statistical evidence to reject the null hypothesis. Based on these results, it can be concluded that at least one specialty exhibits a cancellation rate significantly different from the others. This finding suggests that the type of surgical specialty may play a significant role in determining surgery cancellation rates.

Subsequently, a post-hoc analysis was conducted using the standardized residuals from the chi-square test. Cells with a standardized residual whose absolute value exceeded 1.96 were considered to differ significantly from the expected value, reflecting how far the observed frequency deviated from the expected frequency in terms of standard deviations. In this case, the specialties showing absolute residuals greater than 1.96 were otorhinolaryngology and urology. For otorhinolaryngology, the residual value for cancelled surgeries was 3.4731, indicating a higher frequency of cancellations than expected. For urology, the absolute residual value was 2.69707 for performed surgeries, suggesting a higher-than-expected number of completed procedures. A two-way contingency table was created to display the number of cancellations by specialty and reason (Table 1).

**Table 1:** Number of surgical procedure cancellations by surgical specialty and cancellation reason. LOT: lack of operative time; PNA/W: patient did not attend/withdrew; PRFI: patient-related factors-illness; PRFOR: patient-related factors-other reasons.

Cancellation reason	PRFI	PRFOR	LOT	Strike	PNA/W
Surgical specialty					
General surgery	9 (7.0%)	6 (4.7%)	41 (31.8%)	66 (51.2%)	7 (5.4%)
Vascular Surgery	0 (0%)	0 (0%)	2 (33.3%)	4 (66.7%)	0 (0%)
Stomatology	0 (0%)	2 (50%)	0 (0%)	0 (0%)	2 (50%)
Gynecology	1 (2.0%)	3 (6.1%)	4 (8.2%)	39 (79.6%)	2 (4.1%)
Ophthalmology	9 (8.7%)	5 (4.9%)	4 (3.9%)	82 (79.6%)	3 (2.9%)
Ear, nose, and throat surgery	37 (33.3%)	1 (0.9%)	31 (27.9%)	40 (36.0%)	2 (1.8%)
Orthopedics	4 (2.5%)	8 (4.9%)	54 (33.1%)	88 (54%)	9 (5.5%)
Urology	3 (5.8%)	0 (0%)	16 (30.8%)	31 (59.6%)	2 (3.8%)

A Kruskal-Wallis test (non-parametric ANOVA) was applied to the variables reason for cancellation, surgical specialty, ASA physical status, and month, while the Mann-Whitney U test (Wilcoxon-Mann-Whitney) was used for the variables preoperative consultation and repeat preoperative consultation.

To ensure objective, reproducible, and clinically accurate data extraction, cancellation causes were classified using an adaption of the standardized coding manual of the Integrated Management System for Surgical Waiting Lists (Sistema Integrado de Gestão de Inscritos para Cirurgia-SIGIC). This framework categorizes cancellations into four major operational domains: hospital/administrative responsibility, clinical reasons, patient non-clinical responsibility, and third-party/external factors.

To identify which specific pairs of reasons differed significantly, Dunn's multiple comparison test was performed. Significant differences were found between the following pairs of reasons: (1) “Patient not prepared-illness” vs. “Patient not prepared-other reasons”, (2) “Patient not prepared-illness” vs. “Lack of operative time”, (3) “Patient not prepared-illness” vs. “Strike”, and (4) “Patient not prepared-other reasons” vs. “Patient did not attend/withdrew”.

For the variable surgical specialty, the p-value was also significant (p < 0.001), leading to the rejection of the null hypothesis; i.e., at least one specialty exhibited a median rescheduling time significantly different from the others. A Pearson's chi-square test was performed to assess the association between medical specialty and surgical cancellations.

To identify which specialties differed, Dunn's post-hoc test was again applied, revealing statistically significant differences between the following pairs of specialties: (1) General Surgery and Ophthalmology; (2) General Surgery and Otorhinolaryngology; (3) General Surgery and Urology; (4) Gynecology and Ophthalmology; (5) Ophthalmology and Orthopedics; and (6) Otorhinolaryngology and Orthopedics.

To further examine the relation of preoperative anesthetic consultation variables within each specialty and to explore potential relationships with other study variables, a separate table was created comprising each specialty (Table 2). For qualitative variables, Pearson's chi-square test was used, and for the quantitative variable (rescheduling days), the Wilcoxon-Mann-Whitney test was applied. The specialties stomatology and vascular surgery were excluded due to small sample sizes. The specialties gynecology and urology were not included in Table 2 due to having the lowest cancellation rates.

**Table 2:** Analysis of the variable preoperative anesthetic consultation by surgical specialty. ASA: American Society of Anesthesiologists physical status classification; LOT: lack of operative time; N: no; Y: yes; NA: not applicable; PNA/W: patient did not attend/withdrew; PRFI: patient-related factors-illness; PRFOR: patient-related factors-other reasons.

	General surgery		Orthopedics		Ophthalmology		Ear, nose, and throat surgery	
	Preoperative appointment (Y = yes; N = no)	P-value	Preoperative appointment (Y = yes; N = no)	P-value	Preoperative appointment (Y = yes; N = no)	P-value	Preoperative appointment (Y = yes; N = no)	P-value
Gender								
Male	57 (Y)	p = 0.2362	72 (Y)	p = 1	30 (Y)	p = 0.06335	68 (Y)	p = 0.7202
	10 (N)		9 (N)		23 (N)		1 (N)	
Female	65 (Y)		76 (Y)		22 (Y)		44 (Y)	
	5 (N)		10 (N)		37 (N)		2 (N)	
Age group								
1-4 years	0 (Y)	NA	0 (Y)	NA	8 (Y)	p = 0.001646	22 (Y)	NA
	0 (N)		0 (N)		0 (N)		1 (N)	
5-12 years	0 (Y)		0 (Y)		3 (Y)		47 (Y)	
	0 (N)		0 (N)		0 (N)		0 (N)	
13-65 years	79 (Y)		78 (Y)		10 (Y)		42 (Y)	
	5 (N)		5 (N)		22 (N)		2 (N)	
66-85 years	39 (Y)		12 (Y)		28 (Y)		1 (Y)	
	9 (N)		66 (N)		30 (N)		0 (N)	
>85 years	4 (Y)		4 (Y)		3 (Y)		0 (Y)	
	1 (N)		2 (N)		8 (N)		0 (N)	
Cancellation reason								
Strike	64 (Y)	p = 0.001253	81 (Y)	p = 0.2739	28 (Y)	p = 0.00155	40 (Y)	p < 0.001
	2 (N)		7 (N)		54 (N)		0 (N)	
LOT	36 (Y)		46 (Y)		4 (Y)		29 (Y)	
	5 (N)		8 (N)		0 (N)		2 (N)	
PRFI	5 (Y)		3 (Y)		7 (Y)		37 (Y)	
	4 (N)		1 (N)		2 (N)		0 (N)	
PNA/W	6 (Y)		9 (Y)		0 (Y)		1 (Y)	
	1 (N)		0 (N)		3 (N)		1 (N)	
PRFOR	4 (Y)		6 (Y)		4 (Y)		1 (Y)	
	2 (N)		2 (N)		1 (N)		0 (N)	
ASA physical status								
I	6 (Y)	p = 0.04697	3 (Y)	p = 0.634	9 (Y)	p = 0.03891	45 (Y)	NA
	1 (N)		1 (N)		2 (N)		1 (N)	
II	68 (Y)		89 (Y)		25 (Y)		63 (Y)	
	3 (N)		9 (N)		42 (N)		2 (N)	
III	43 (Y)		51 (Y)		16 (Y)		4 (Y)	
	8 (N)		7 (N)		14 (N)		0 (N)	
IV	4 (Y)		4 (Y)		2 (Y)		0 (Y)	
	2 (N)		0 (N)		2 (N)		0 (N)	
Rescheduling days								
Mean ± standard deviation	22.4 ± 25.4 (Y)	p = 0.02674	25.4 ± 29.8 (Y)	p < 0.001	42.7 ± 34.9 (Y)	p = 0.2358	30.9 ± 25.5 (Y)	p = 0.1252
	13.4 ± 19.1 (N)		4.75 ± 5.30 (N)		35.5 ± 26.9 (N)		10.5 ± 4.95 (N)	

## Results

Descriptive information

To provide a general description of the dataset, Table 3 presents the sociodemographic characteristics of the sample. The table includes qualitative variables, displayed with their respective number of observations and percentages, and one quantitative variable, “Days between scheduled surgery date and rescheduled date,” presented with the corresponding mean and standard deviation (SD).

**Table 3:** Sociodemographic variables and respective cancellation frequencies. ASA: American Society of Anesthesiologists physical status classification; LOT: lack of operative time; OR: operating room; PNA/W: patient did not attend/withdrew; PRFI: patient-related factors-illness; PRFOR: patient-related factors-other reasons; SD: standard deviation.

Sociodemographic variables	Qualitative variables
Gender	
Male	316 (48.8%)
Female	331 (51.2%)
Age group	
1-4 years	31 (4.8%)
5-12 years	51 (7.9%)
13-65 years	320 (49.4%)
66-85 years	220 (34%)
>85 years	25 (3.9%)
Surgical specialty	
General surgery	137 (21.2%)
Vascular surgery	6 (0.9%)
Stomatology	4 (0.6%)
Gynecology	50 (7.7%)
Ophthalmology	112 (17.3%)
Ear, nose, and throat surgery	115 (17.8%)
Orthopedics	167 (25.8%)
Urology	56 (8.7%)
Reason for cancelling	
Strike	350 (54%)
Lack of operative time (LOT)	152 (23.3%)
Patient-related factors-illness (PRFI)	63 (9.7%)
Patient-related factors-other reasons (PRFOR)	25 (3.9%)
Patient did not attend/withdrew (PNA/W)	27 (4.2%)
Others: critical accident in or; non-confirmed diagnosis; anesthetic risk-surgical risk; lack of ancillary diagnostic tests; medical record error; lack of conditions in or; lack of material; lack of postoperative bed; surgeon absence	1 (0.2%); 4 (0.6%); 5 (0.8%); 6 (0.9%); 3 (0.5%); 1 (0.2%); 1 (0.2%); 1 (0.2%); 5 (0.8%); 3 (0.5%)
ASA physical status	
I	85 (13.1%)
II	370 (57.2%)
III	171 (26.4%)
IV	11 (1.7%)
No record	10 (1.5%)
Month	
January	29 (4.5%)
February	55 (8.5%)
March	95 (14.7%)
April	36 (5.6%)
May	55 (8.5%)
June	91 (14%)
July	99 (15.3%)
August	86 (13.3%)
September	28 (4.3%)
October	73 (11.3%)
Preoperative anesthetic consultation	
Yes	536 (82.8%)
No	111 (17.2%)
Subsequent preoperative anesthetic consultation	
Yes	11 (1.7%)
No	636 (98.3%)
	Qualitative variables (mean ± SD)
Days: scheduled surgery date-rescheduled date	28.42 ± 28.87

With respect to surgical specialties, the highest cancellation frequencies were observed in orthopedics (25.8%, n = 167), general surgery (21.2%, n = 137), otorhinolaryngology (17.8%, n = 115), and ophthalmology (17.3%, n = 112). Conversely, the specialties with the fewest cancellations were stomatology (0.6%, n = 4) and vascular surgery (0.9%, n = 6).

Considering the reasons for cancellation, the five most frequent were: strike (54%, n = 350), lack of operative time (LOT) (23.3%, n = 152), patient-related factors-illness (PRFI) (9.7%, n = 63), patient did not attend/withdrew (PNA/W) (4.2%, n = 27), and patient-related factors-other reasons (PRFOR) (3.9%, n = 25). All remaining causes, each accounted for less than 1% of cancellations.

In terms of ASA physical status, ASA II was the most common classification (57.2%, n = 370), followed by ASA III (26.4%, n = 171). Cases categorized as “no record” corresponded to patients whose ASA status was not available.

Across the 10-month study period, the months with the highest number of cancellations were July (15.3%, n = 99), March (14.7%, n = 95), June (14.0%, n = 91), August (13.3%, n = 86), and October (11.3%, n = 73).

The number of cancellations involving patients who attended a preoperative anesthetic consultation was considerably higher than those who did not, with 82.8% (n = 536) and 17.2% (n = 111), respectively. In contrast, only 1.7% (n = 11) of the cancellations were associated with a subsequent preoperative anesthetic consultation, while 98.3% (n = 636) were not.

For the quantitative variable “days between scheduled and rescheduled surgery,” the mean was 28.42 days with a standard deviation of 28.87 days. The standard deviation exceeding the mean likely reflects the heterogeneity of the data and the presence of multiple outliers (extreme values).

For subsequent analyses, only the five most frequent reasons for cancellation (as listed above) were considered to enhance the feasibility, quality, and interpretability of the statistical analyses and data visualization.

Local cancellation rate

Between January 1 and October 31, 2023, a total of 4637 elective surgeries were performed, out of 5284 scheduled procedures. Given that 647 elective surgeries were cancelled (sample size), the elective surgery cancellation rate was 12.24%.

Subgroup analyses of cancellation rate by surgical specialty and most frequent reasons

Similarly, the cancellation rate for each surgical specialty was calculated, as presented in Table 4.

**Table 4:** Cancellation rate by surgical specialty.

Surgical specialty	Number of surgeries performed	Number of cancelled surgeries	Total number of scheduled surgeries	Cancellation rate (%)	P-value
General surgery	884	137	1021	13.42	p < 0.001
Vascular surgery	22	6	28	21.43	
Stomatology	12	4	16	25	
Gynecology	457	50	507	9.86	
Ophthalmology	782	112	894	12.53	
Ear, nose, and throat surgery	597	115	688	16.72	
Orthopedics	1333	167	1500	11.33	
Urology	574	56	630	8.89	

The specialty with the highest cancellation rate was stomatology (25.0%), followed by vascular surgery (21.43%). Conversely, the specialties with the lowest cancellation rates were urology (8.89%) and gynecology (9.86%). Across all specialties, strike was the most common reason for cancellation, except in stomatology, where no cancellations were attributed to this cause. Notably, gynecology and ophthalmology had particularly high percentages of cancellations due to strike (79.6% in both). In otorhinolaryngology, the proportion of cancellations attributed to “patient not prepared-illness” was nearly as high as that due to strike and “lack of operative time”. Thus, unlike other specialties, there was no marked discrepancy between the frequencies of these causes within this specialty. Table 5 presents the mean number of days to rescheduling for each variable, along with the respective standard deviation.

**Table 5:** Rescheduling days (days of delay from the scheduled intervention date to the rescheduled date), on average, for each variable, with the respective standard deviation. ASA: American Society of Anesthesiologists physical status classification; LOT: lack of operative time; PNA/W: patient did not attend/withdrew; PRFI: patient-related factors-illness; PRFOR: patient-related factors-other reasons; SD: standard deviation.

	Rescheduling days (mean ± standard deviation)	P-value
Cancellation reason		
PRFI	43.60 ± 29.67	p < 0.001
PRFOR	28.35 ± 40.43	
LOT	20.14 ± 25.26	
Strike	27.96 ± 26.22	
PNA/W	69.50 ± 44.15	
Surgical specialty		
General surgery	23.0 ± 28.4	p < 0.001
Vascular surgery	64.0 ± 54.0	
Stomatology	49.0 ± 0.00	
Gynecology	25.8 ± 30.4	
Ophthalmology	38.9 ± 30.4	
Ear, nose, and throat surgery	30.6 ± 25.3	
Orthopedics	23.3 ± 28.8	
Urology	29.1 ± 20.1	
ASA physical status		
I	34.9 ± 30.4	p < 0.01
II	28.0 ± 27.9	
III	25.7 ± 29.0	
IV	15.8 ± 18	
Preoperative anesthetic consultation		
Yes	28.3 ± 28.5	p = 0.3208
No	29.1 ± 30.9	
Subsequent anesthetic consultation		
Yes	30.6 ± 55.1	p = 0.2405
No	28.4 ± 28.4	
Month		
January	32.4 ± 32.6	p = 0.03025
February	22.3 ± 21.3	
March	31.6 ± 34.4	
April	19.9 ± 17.5	
May	25.1 ± 32.7	
June	34.3 ± 36.4	
July	31.8 ± 30.1	
August	31.8 ± 24.8	
September	22.3 ± 17.9	
October	19.3 ± 13.9	

Subgroup analyses of cancellation rate by American Society of Anesthesiologists (ASA) physical status and preoperative anesthetic consultation

Regarding the ASA physical status classification, statistically significant differences in the median number of rescheduling days were present between ASA Class I and Class III.

For the variable preoperative consultation, the Mann-Whitney U test produced a p-value of 0.3208 (greater than 0.05), indicating that the null hypothesis of independence between the variables consultation and rescheduling days cannot be rejected. Thus, there is no statistically significant association between undergoing a pre-anesthetic consultation and the number of days required to reschedule surgery. The same result was obtained for the variable new consultation, with a p-value of 0.2405 (Mann-Whitney U test), again suggesting no statistically significant association between having a new preoperative consultation and the delay in rescheduling.

For the last variable presented in the table, the month of intervention, the Kruskal-Wallis test was applied to evaluate the existence of significant differences in rescheduling days among months. A significant p-value (0.03025) was obtained, indicating statistical evidence that at least one month differs significantly in terms of rescheduling days. Dunn's test was then conducted to identify which pairs of months differed, revealing a statistically significant difference in rescheduling days between August and October.

Continuing with the topic of rescheduling days, particularly regarding their relationship with the month of intervention, as noted previously, the months with the highest number of cancellations were July, March, June, August, and October, while January (4.5%, n = 29) and April (5.6%, n = 36) had the lowest numbers.

Further analysis of the intervention month and the average number of days to reschedule surgeries per month. The month of June showed the highest mean (34.35 days), followed by January (32.43 days), July (31.85 days), August (31.77 days), and March (31.62 days). Conversely, the months with the lowest averages were October (19.26 days), April (19.86 days), February (22.31 days), and September (22.32 days).

From the observation of these data, two noteworthy situations emerge: January had one of the lowest numbers of cancellations but one of the highest average rescheduling times, whereas October had a high number of cancellations but the lowest mean rescheduling time. These discrepancies may be explained by outliers in the data.

Analyzing the distribution of the five most frequent reasons for cancellation across the study months, it is evident that strike was the most frequent reason for cancellation in all months where it occurred (February, March, May, June, July, August, and October), except in May, when cancellations due to lack of operative time surpassed those due to strike. The month with the highest number of strike-related cancellations was July.

Starting with general surgery, the p-value for the variable gender was not significant, indicating no statistical evidence of an association between preoperative consultation and patient gender in this specialty. For the variable reason for cancellation, a significant p-value of 0.001253 was obtained, suggesting a statistically significant association between preoperative consultation and reason for cancellation in general surgery. Similarly, for the variable ASA physical status, the p-value was 0.04697 (significant), indicating a significant association between these two variables. For rescheduling days, the Wilcoxon-Mann-Whitney test yielded a significant p-value of 0.02674, suggesting a statistically significant difference in the distributions of preoperative consultation and rescheduling days within general surgery.

In orthopedics, the only variable with a significant p-value was rescheduling days (p < 0.001), indicating a significant difference between patients who had or did not have a preoperative consultation in terms of rescheduling delay. No statistically significant associations were found for the remaining variables.

In ophthalmology, the variables gender and rescheduling days did not show a significant association with having a pre-anesthetic consultation. However, significant p-values were obtained for the variables age group, reason for cancellation, and ASA classification, indicating statistically significant associations with preoperative consultation.

Finally, in otorhinolaryngology, the only variable with a significant p-value was the reason for cancellation (p < 0.001), demonstrating statistical evidence of a significant association between reason for cancellation and preoperative consultation in this specialty.

## Discussion

During the study period, 4,637 elective surgeries were performed, of which 647 were cancelled, resulting in an overall cancellation rate of 12.24%, suggesting that a substantial proportion of surgeries were affected by cancellations. This figure aligns well with reported elective surgery cancellation rates in international literature, which generally range between 1% and 24% in high-income healthcare settings [7,11].

The analysis of cancellations by surgical specialty revealed significant differences in cancellation rates. Stomatology and vascular surgery showed the highest rates, 25.0% and 21.43%, respectively, although these figures were based on small absolute numbers (four and six cancellations, respectively), indicating potential statistical volatility. Conversely, urology and gynecology exhibited the lowest cancellation rates, 8.89% and 9.86%, respectively.

To assess the association between medical specialty and surgical cancellations, the results provided significant statistical evidence to reject the null hypothesis, suggesting that medical specialty plays a significant role in surgical cancellation rates. The post-hoc analysis identified otorhinolaryngology and urology as potential contributors to this association. The absolute values of standardized residuals in these specialties indicate that observed frequencies differed significantly from expected ones, suggesting that these specialties have a distinct impact on cancellation rates.

These specialty-specific disparities mirror previous multicenter findings by Leslie et al. and Schuster et al. [6,11], who noted that high-volume surgical services with complex emergency-elective overlapping workflows, such as general surgery and orthopedics, consistently experience greater schedule volatility.

Regarding the reasons for cancellation by specialty, strike action emerged as the most common cause across all specialties, except in stomatology. This finding suggests that external factors, such as strikes, exert a substantial influence on surgical cancellations overall.

While most literature categorizes primary cancellation drivers into administrative, clinical, or patient-related factors [1,10], external systemic factors can temporarily overshadow internal hospital metrics.

Notably, in gynecology and ophthalmology, the proportion of cancellations due to strikes was particularly high (79.6%), indicating a potential vulnerability of these specialties to external disruptions. In otorhinolaryngology, the proportion of cancellations due to patients being unprepared because of illness was almost as high as that due to strikes, and lack of operating time was also a significant factor. This suggests that, in this specialty, patient-related factors and operative time management play crucial roles in cancellations.

Excluding strikes, "lack of operative time" (23.3%) emerged as the primary modifiable institutional cause of cancellation. This finding is widely corroborated across the perioperative literature, where overbooking, unexpected intraoperative extensions, and emergency case interruptions are cited as the leading operational causes of same-day cancellations [12,14].

In summary, results on reasons for elective surgery cancellations by specialty show that external factors, particularly strikes, play a major role. Furthermore, variation in reasons across specialties highlights the need for specialty-specific strategies to reduce cancellations and improve overall surgical workflow efficiency.

The variables reason for cancellation, specialty, ASA physical status, and month of intervention all influenced the mean number of days to reschedule surgery. The findings suggest that patients classified as ASA I and ASA III had significantly different mean rescheduling times.

Interestingly, patients classified as ASA I experienced longer average rescheduling delays than ASA III patients (34.9 vs. 25.7 days). This suggests that clinical triage practices prioritize higher-acuity patients with greater physiological vulnerability for expedited rescheduling, consistent with risk-stratified perioperative management guidelines [15].

The presence of an anesthetic preoperative consultation or a new preoperative consultation was not statistically associated with rescheduling days. These results underscore the importance of considering specific factors, such as cancellation reason and specialty, when managing surgical rescheduling.

The months with the highest number of cancellations were July, March, June, August, and October, while January and April had the fewest. This pattern may reflect seasonal trends or specific events associated with peaks in cancellations during certain times of the year. January was particularly noteworthy for having one of the lowest numbers of cancellations but one of the highest mean rescheduling times. This phenomenon may be explained by individual cases with long rescheduling delays, suggesting the presence of outliers in the data. A more detailed analysis of these individual cases is warranted to better understand the underlying causes of prolonged rescheduling intervals.

The analysis of the preoperative anesthetic consultation variable across specialties emphasized its potential relevance in the surgical process. The statistical associations observed suggest that the presence or absence of a preoperative consultation may influence various aspects of the surgical process, ranging from the reason for cancellation to the rescheduling delay. The differences observed among specialties highlight the importance of tailored approaches to managing preoperative consultations according to each clinical context.

A central finding of this study was that strikes accounted for over half (54.0%) of all elective surgery cancellations during the observation period. It is crucial to contextualize this figure as a temporary, exceptional circumstance rather than a recurring structural inefficiency within the hospital's operating theater framework. The study period (January-October 2023) coincided with an extraordinary sequence of nationwide healthcare sector strikes in Portugal driven by National Labor Union negotiations involving physicians and nursing staff. These events particularly impacted scheduled elective lists.

However, it is important to acknowledge several study limitations and potential biases. One limitation concerns the study period, as only a few months were analyzed; a longer observation period could have provided a more representative sample and more robust findings. Additionally, the limited timeframe restricts the ability to identify long-term patterns or seasonal variations. Moreover, because the study relied on a census-based sample of convenience within this fixed timeframe, no a priori power calculation was conducted.

Another limitation is disparities in sample sizes across surgical specialties, particularly in low-volume specialties (stomatology and vascular surgery), which yielded wide confidence intervals and high rate volatility; as a result, inter-specialty rate comparisons cannot serve as robust baseline benchmarks and should be treated as purely exploratory.

Furthermore, since this was an observational study conducted in a single hospital, the results and conclusions cannot be directly generalized to other healthcare institutions with different organizational or contextual characteristics. A standardized definition and classification system for surgical cancellations would facilitate comparative analysis across institutions and improve the overall consistency of future research on this topic.

## Conclusions

Among all elective surgeries scheduled at ULSRA during the study period, the cancellation rate obtained was consistent with international benchmarks, which underscores the relevance of the issue. Strike action was the leading cause of cancellation, reflecting an exceptional period of nationwide healthcare strikes rather than a structural inefficiency. Excluding strikes, lack of operative time was the main modifiable cause.

Cancellation rates varied significantly by surgical specialty, with the highest numbers being observed in orthopedics. In otorhinolaryngology, patient-related factors and operative time management played a crucial role in cancellations. The rescheduling delay was influenced by cancellation reason, surgical specialty, ASA physical status, and month of intervention, but not by preoperative anesthetic consultation. The months of July, March, June, August, and October recorded the highest numbers of cancellations, suggesting possible seasonal patterns or specific temporal events contributing to these peaks. These findings support the development of specialty-tailored strategies to reduce cancellations, particularly targeting operative time management, and highlight the need for multi-year, multicenter studies with standardized cancellation definitions to confirm and generalize these results. Another valuable approach would be to include an economic analysis, examining the financial impact of cancellations, including costs associated with wasted resources and surgical schedule reorganization.
